# The Federation of Medical and Allied Societies

**Published:** 1921-07-02

**Authors:** 


### The Federation of Medical and Allied Societies.

The annual general meeting was held at 11 Chandos Street, London, W. 1, on Tuesday, June 28.

In the unavoidable absence of the President, Sir Berkeley Moynihan, the Vice-President, Sir Malcolm Morris, delivered the annual address. The annual report of the Executive Council was received and adopted. The President, Vice-President, Hon. Treasurers, and Auditor were re-appointed for the ensuing year, and thirty Societies were elected to representation on the Executive Council. The result of the ballot for a representation of individual members was declared in favour of Dr. Ford Anderson.

The meeting of the Executive Council was held before the annual meeting, Sir Malcolm Morris being in the chair. A report was received from the Medical Council concerning the question of pressing for an inquiry into the medical provisions of the National Insurance Acts, especially with a view to adding certain services now lacking. The General Purposes Committee reported on a reference it had received regarding the publication of the news of the Federation, and it was resolved to issue a journal, the arrangements being left in the hands of a Special Committee appointed for the purpose. It was decided not to recommend an increase in the rates of subscriptions from Societies. The transfer of the offices to new premises at 12 Stratford Place was authorised.

